# Regeneration of the power performance of cathodes affected by biofouling

**DOI:** 10.1016/j.apenergy.2016.04.009

**Published:** 2016-07-01

**Authors:** Grzegorz Pasternak, John Greenman, Ioannis Ieropoulos

**Affiliations:** a Bristol BioEnergy Centre, Bristol Robotics Laboratory, University of the West of England, Coldharbour Lane, BS16 1QY Bristol, UK; b Wroclaw University of Technology, Wyb. Wyspianskiego 27, 50-370 Wroclaw, Poland

**Keywords:** Biodeterioration, Microbial fuel cell, Air cathode, Biofilm, Fouling, Lysis

## Abstract

Air cathode microbial fuel cells (MFCs) were used in a cascade-system, to treat neat human urine as the fuel. Their long-term operation caused biodeterioration and biofouling of the cathodes. The cathodes were made from two graphite-painted layers, separated by a current collector. The initial performance of the MFCs was reaching average values of 105.5 ± 32.2 μW and current of 1164.5 ± 120.2 μA. After 3 months of operation the power performance decreased to 9.8 ± 3.5 μW, whilst current decreased to 461.2 ± 137.5 μA. Polarisation studies revealed significant transport losses accompanied by a biofilm formation on the cathodes. The alkaline lysis procedure was established to remove the biomass and chemical compounds adsorbed on the cathode’s surface. As a result, the current increased from 378.6 ± 108.3 μA to 503.8 ± 95.6 μA. The additional step of replacing the outer layer of the cathode resulted in a further increase of current to 698.1 ± 130 μA. Similarly, the power performance of the MFCs was recovered to the original level reaching 105.3 ± 16.3 μW, which corresponds to 100% recovery. Monitoring bacterial cell number on the cathode’s surface showed that biofilm formed during operation was successfully removed and composed mainly of dead bacterial cells after treatment. To the best of the authors’ knowledge, this is the first time that the performance of deteriorating cathodes, has been successfully recovered for MFCs *in-situ*. Through this easy, fast and inexpensive procedure, designing multilayer cathodes may help enhance the range of operating conditions, if a biofilm forms on their surface.

## Highlights

• Detrimental biofilm growth on cathodes has been successfully removed, allowing full power recovery.
• An *in-situ* chemical regeneration method, for bio-fouled cathodes is proposed.
• Introducing multiple sacrificial layers for cathode electrodes, can be a good recovery mechanism.

## Graphical abstract

**Figure:**
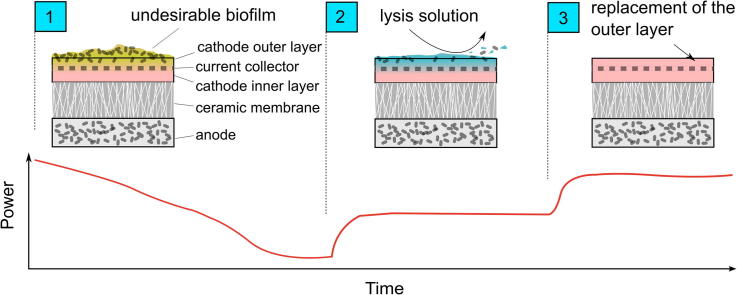

## 1 Introduction

Microbial Fuel Cell (MFC) is a bioelectrochemical reactor in which electroactive bacteria convert the energy stored in various chemical substrates, into electricity [1]. A number of factors are influencing the overall MFC performance and complex mathematical models have been proposed to describe these systems [2].

The MFC technology is a sustainable way of producing energy, which has been used in a variety of applications, the most common of which include treating wastewater and nutrient recovery [3], [4]. It was also shown that urine can be successfully treated in MFCs [5], and in some other examples, H_2_ production has been reported [6]. Treating urine in MFCs was recently scaled up to pilot scale [7]. Our studies showed that power was sufficient to power LED lights and that urea was not contributing to the generation of power. So far, successful transformation of urea into electrical energy was only demonstrated in conventional fuel cells, including solid oxide fuel cells [8]. An interesting example of a hybrid technique using MFCs was described by Chen et al. who reported an electrodialysis system, consisting of an MFC used for alkali production and demonstrated its use for biogas upgrading [9]. Bioelectrochemical systems (BESs) can also be employed to techniques for sustainable synthesis of chemicals in a process known as microbial electrosynthesis [10], as well as for the recovery of valuable metals by reduction on the anode surface as demonstrated by Wang et al. [11]. The cathodic reactions may also lead to the removal of toxic hexavalent chromium as reported by Xafenias et al. [12].

Regardless of the design and application, the essential parts of every MFC are the anode and the cathode. These elements determine the overall performance of the MFC and both are susceptible to dynamic changes taking place during their operation. These changes include increasing the biofilm thickness as well as precipitation and adsorption of chemical compounds on the electrode surface [13], [14]. The biofilm activity on the cathode was widely used as an approach for increasing the oxygen reduction rates, when pure phototrophic cultures or aerobic activated sludge were used as the biocatalyst [15], [16]. Similarly, the utilisation of the laccase enzyme, an enzyme synthesised by several fungal species, which reduces O_2_ to H_2_O, leads to improved performance and extends the lifetime of the cathode [17], [18]. On the other hand, unwanted biofilm growth on the surface of MFC components such as the proton exchange membrane or the cathode, may deteriorate the performance due to biofouling [19], [20].

In recent studies workers have indicated that a power reduction during long-term MFC operation may be caused by biofouling or fouling of the membrane or the cathode. Behera et al. [21] observed a power drop just after 35 days of operation in ceramic MFCs. Pasternak et al. [22] reported decrease of performance after 32 days of operation of air–cathode MFCs built from four different types of ceramic materials. Similar observations were also reported by other authors, even when other types of material have been used to construct MFCs [23], [24]. However, none of these studies have scientifically proven that it is the biofouling of the cathode that causes the power drop [21], [22], [24], [25], [26], [27]. Chung et al. [23] reported that deterioration of the cathode was accompanied by the biofilm formation. However, following the cleaning procedure (10% HCl for one hour), the performance of the cathode decreased further. This phenomenon was also investigated by Yuan et al. [28] and it was found, that the presence of a biofilm on the inner (anode facing) layer of the cathode may decrease the performance of the MFC. Nevertheless, a solution for combating the undesirable biofilm growth has not yet been proposed.

Although the presence of a biofilm on the outer layer of the cathode can be inspected at a macroscopic scale, it is important to determine whether this biofilm may cause the power drop in MFCs. In the field of conventional fuel cells science, R&D has led to determining the role of cathode deterioration on power performance and appropriate strategies are currently being developed to address this problem [29], [30].

In the field of MFCs, several long-term studies have reported deteriorating performance levels, primarily due to challenges with setup [31], environmental/experimental conditions and material degradation [24], [32]. In particular, cathode biofouling has been reported as one of the main factors for decreased performance [23], [24]. Investigating the impact of the undesirable biofilm growth on the cathodic performance, preventing it, or combating it once it occurs, is essential for the design and long-term operation of MFC-based scaled-up systems and their implementation into real world problems.

In this paper we investigated the characteristics of a serious performance deterioration observed for open-to-air cathode MFCs, operated for 131 days. The aim of this study, in addition to identifying the reasons for the significant power reduction, was to find a fast and simple method for *in-situ* regeneration of the conductive graphite-painted cathodes and recover their performance to the original levels.

## 2 Materials and methods

### 2.1 MFC construction and operation

Small scale, single chamber continuous flow MFCs (SCMFCs) were built using ceramic earthenware cylinders as both the chassis and the proton exchange membrane (Scientific & Chemical Supplies Ltd, UK). The ceramic tubes were cut to maintain the internal volume of empty MFCs equal to 11.4 mL. The anodes were made of carbon fibre veil and the cathodes were applied using conductive paint. For the carbon veil anode, the carbon loading was 20 g/m^2^ (PRF Composite Materials, Dorset, UK). Carbon veil was cut into rectangles and pierced with a plain NiCr wire (Ø0.45 mm, Scientific Wire Company, UK). The total surface area of the anode was 252 cm^2^. For the cathode, the outer ceramic membrane surface was covered with a conductive graphite paint prepared as described by Winfield et al. [33]. In brief the polyurethane rubber coating (PlastiDip, Petersfield, UK) was dissolved in petroleum spirit and mixed with graphite (Fisher Chemicals, UK) in a 1:3 (plastidip:graphite) ratio. After covering the ceramics with the first layer (carbon loading of 21.12 mgC/cm^2^) a stainless steel mesh was used as the current collector (20 × 20, 0.18 mm). The mesh was then covered with an additional layer corresponding to a carbon loading of 14.08 mgC/cm^2^. The projected surface area was 24.18 cm^2^ and the total carbon loading for the whole electrode was 0.851 gC. The two layers of the cathode were separated by the current collector to decrease the internal resistance of the electrode. The stainless steel mesh allowed better integrity of the MFC as a whole and the thin, outer layer of the cathode was crucial to improve the contact between the current collector and the conductive carbon material.

**Fig. 1:**
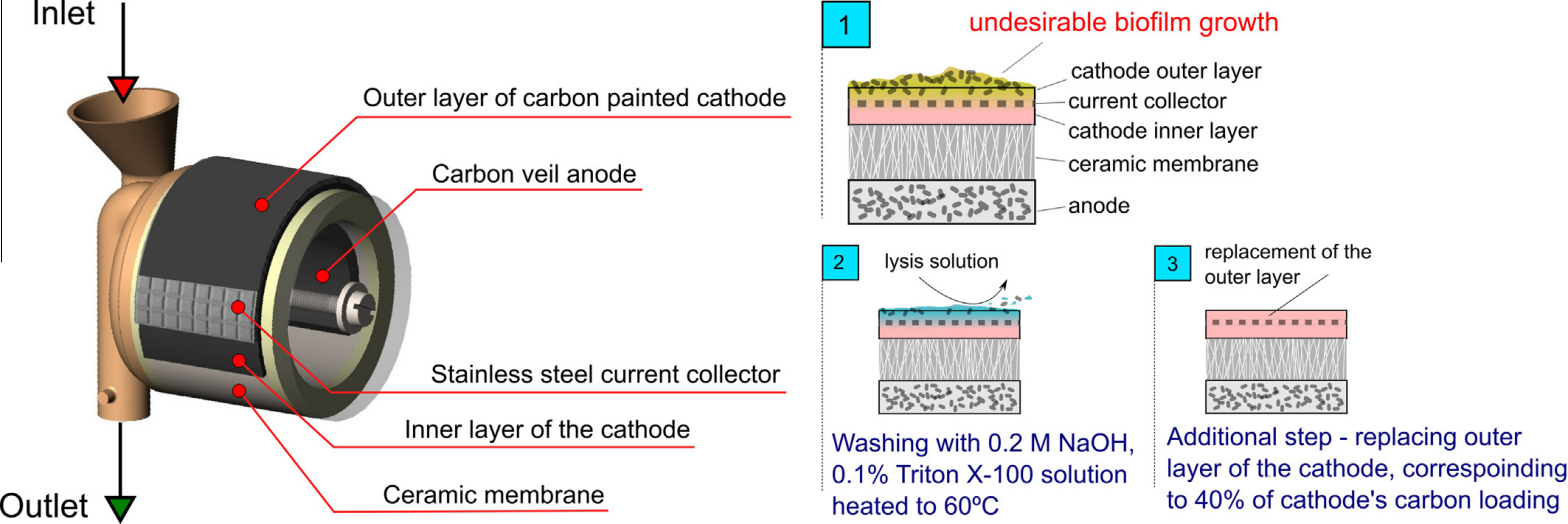
Schematic representation of the MFC design and the principle of regeneration procedure. The cascade consisted of 9 individual MFCs. All of them were subjected to the regeneration procedure.

The MFCs comprised a 3D printed Nanocure® RCP30-resin lid with inlet and outlet tubes, and a transparent acrylic lid (3 mm thick). SolidWorks 2013 software was used to design the RCP30 lids, which were manufactured with Perfactory4 3D printer (Envisiontec, Germany). Silicon gaskets were used to seal the space between the acrylic and RCP30 lids. Both lids were attached to the ceramic membranes by a single plain nylon screw (Ø3 mm, RS, UK). The MFC design is shown in Fig. 1.

A cascade of 9 MFCs was operated in continuous flow conditions. To avoid a liquid electrically conductive bridge between the units, the cells were fluidically isolated by gas gap fluid drip mechanisms – a physical air gap between the MFCs. The MFCs were inoculated with electroactive bacteria derived from existing MFCs with activated sludge. The external load connected to each MFC, was 1000 Ω for the initial 11 days of operation and 250 Ω afterwards. The cascade was fed with fresh human urine as the fuel. The fuel was supplied in a continuous flow mode using a multichannel peristaltic pump (Watson Marlow, USA) at a flow rate of 0.12 L/d, unless otherwise stated.

### 2.2 Polarisation experiments

Polarisation experiments were performed in order to characterise the MFC performance. This was done before and after the power decrease as well as after the regeneration of the cathodes. The fully automated variable resistor system, known as the resistorstat [34] was used. The range of resistors for the polarisation run was 1 MΩ–3.75 Ω, and each value was connected for a period of 5 min.

### 2.3 Regeneration of the cathodes

The first step of regeneration consisted of washing the surface of the cathodes with 2 mL of 0.1 M NaOH solution. Subsequently, washing was repeated with the lysis solution (0.2 M NaOH, 0.1% Triton X-100) heated to 60 °C. The washing was performed by applying the solution slowly at the outer layer of the cathode starting from the top of the MFC (Fig. 1). The excess solution was collected at the bottom of the MFC and the residual solution was left on the cathode for 1–2 min. After each step, the cathodes were washed with de-ionised water to minimise the impact of an increased pH level on the performance of the cathode. The second step of regeneration consisted of removing the external layer of the conductive carbon paint (CCP) and re-painting it *in-situ* with a new layer of identical carbon loading. The procedure is shown in Fig. 1.

An alternative regeneration procedure was performed on individual MFCs operated in batch conditions. The regeneration procedure consisted solely of chemical treatment of the cathodes. The cathode was washed with 2 mL of lysis solution and rinsed with deionized water afterwards. The procedure was repeated until stable power output was recorded and no further increase of performance was observed.

### 2.4 Environmental scanning electron microscopy (ESEM)

Biofilm presence at the cathode was investigated by field emission ESEM (Philips XL-30). Samples of the biofilm were collected from the cathode and fixed with 4% glutaraldehyde in 0.1 M PBS buffer. Subsequently, the samples were rinsed with water and air-dried.

### 2.5 Flow cytometry

The efficiency of the regeneration procedure against the active bacterial biomass was investigated using live/dead bacteria staining and flow cytometry techniques. The cathode samples were collected from individual MFC (2^nd^ MFC in the cascade) and stored in -20 °C until analysis. Samples were taken from: new cathodes from MFCs operating for 2 weeks; biofouled cathodes from MFCs operating for 3 months and regenerated cathodes, operating for 1 month following regeneration. The cathodic material was centrifuged and resuspended in 0.2 μm – filtered NaCl solution (0.85%) and vortexed for at least 1 min. Dilutions and flow cytometry analysis were made as described before [22].

### 2.6 Data logging and processing

The performance of the MFCs was recorded using a Picolog ADC-24 Data Logger (Pico Technologies, UK), with the data logging sample rate set to 3 min. The current was calculated according to Ohm’s law: *I* = *V*/*R*, where *V* is the measured voltage in Volts (V) and *R* is the value of the external resistance. The power output *P* in Watts (W) was calculated using equation: *P* = *I* × *V*. Experimental data were processed using Microsoft Excel 2010 and plotted using the GraphPad Prism software package.

## 3 Results and discussion

### 3.1 Biofouling and reduction of the initial power

**Fig. 2:**
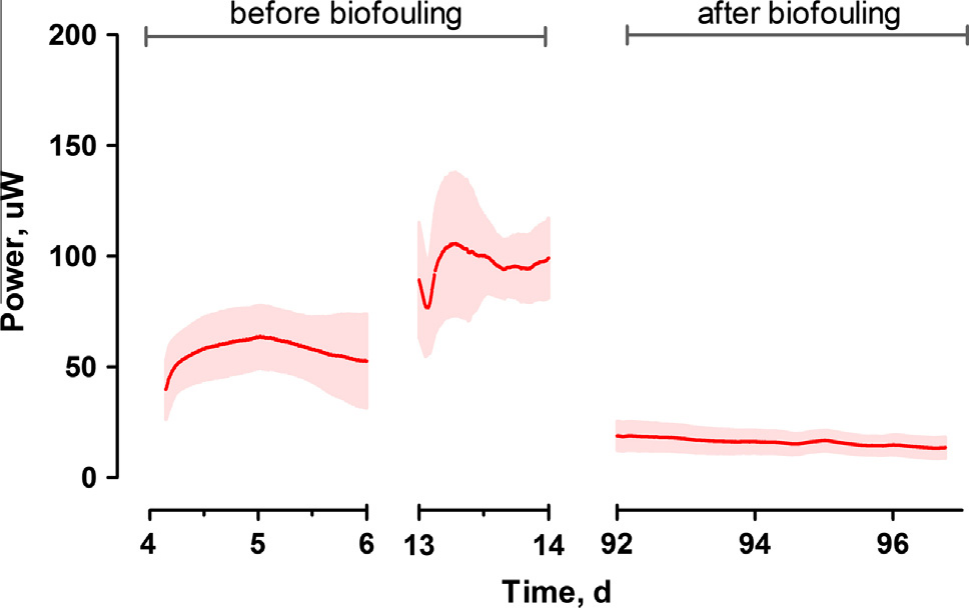
Temporal performance of MFCs before the removal of the cathodic biofilm. Data represent average ± standard deviation. Data recorded for all MFCs in the cascade.

**Fig. 3:**
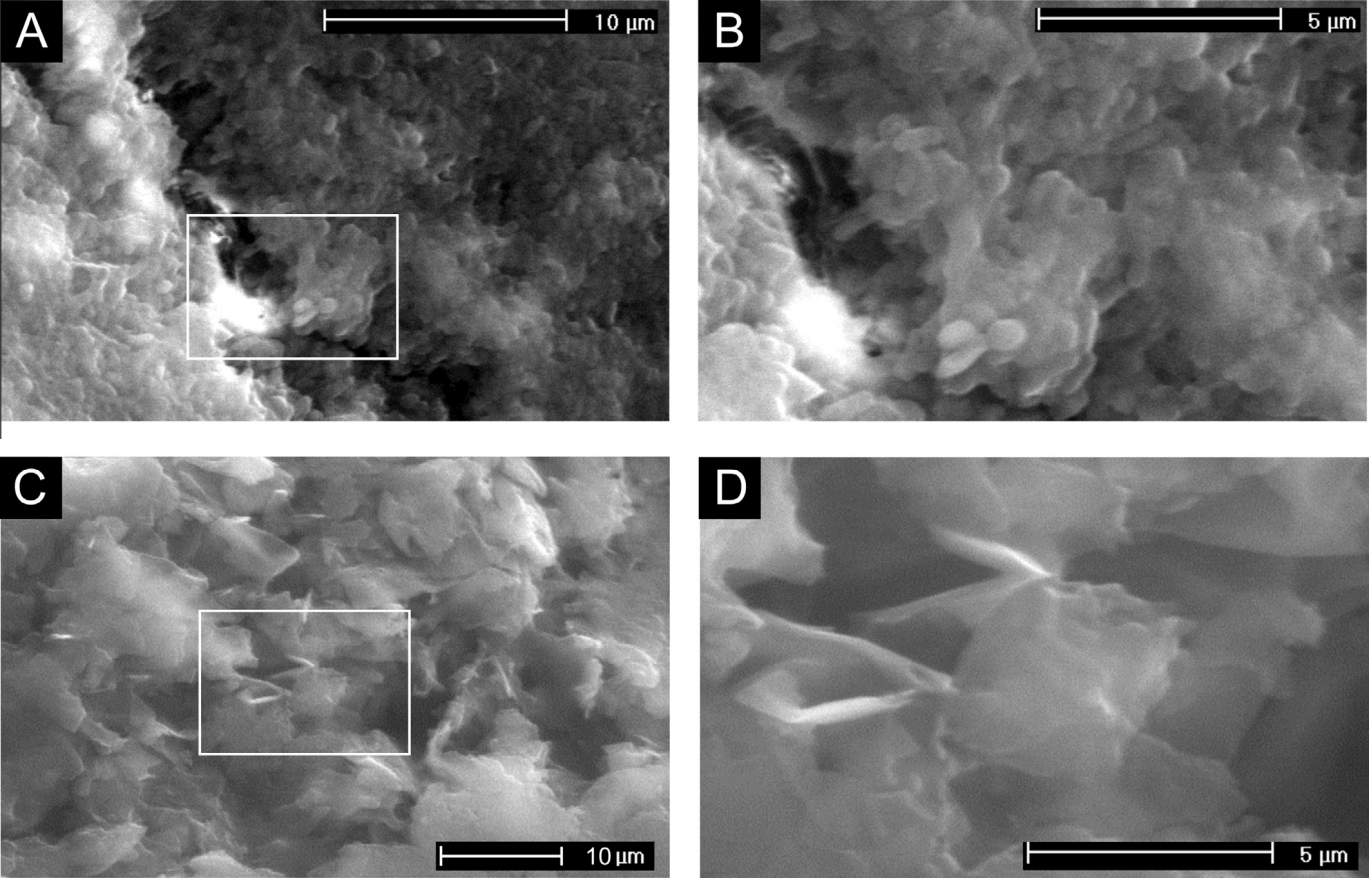
Environmental scanning electron microscopy (ESEM) micrographs of the individual cathode affected by biofouling: before (A and B) and after (C and D) chemical regeneration. The white rectangles represent the area that was image-processed (B, D) in higher magnification. Samples were taken from the 2^nd^ MFC in the cascade. Significant amounts of bacterial cells and exopolymeric substances (EPS) can be observed on the cathode before regeneration.

The cascade of MFCs was operating for 2 months before a significant power reduction was observed. The highest observed power performance values, extracted from the polarisation experiments, reached 150 μW, with average real time values of 105.5 ± 32.2 μW. During the operation of the cascade, biofilm formation was observed on the cathode electrode surface. Its bacterial presence was related to the possible diffusion of organic compounds through the ceramic pores and leaking through the silicone gaskets. After 3 months of continuous operation, the overall performance decreased to values as low as 9.8 ± 3.5 μW (Fig. 2). The biofilm formation was not observed for the open circuit control MFCs, suggesting that the electron flow with the concomitant cation transport through the ceramic membrane, (which inevitably carries water molecules), made the cathodic conditions more favourable to biofilm growth. The power reduction of individual MFCs (Fig. 2) was observed in parallel with the biofilm formation and the corresponding presence of the extracellular polymeric substances (Fig. 3).

### 3.2 Regeneration of the cathodes

**Fig. 4:**
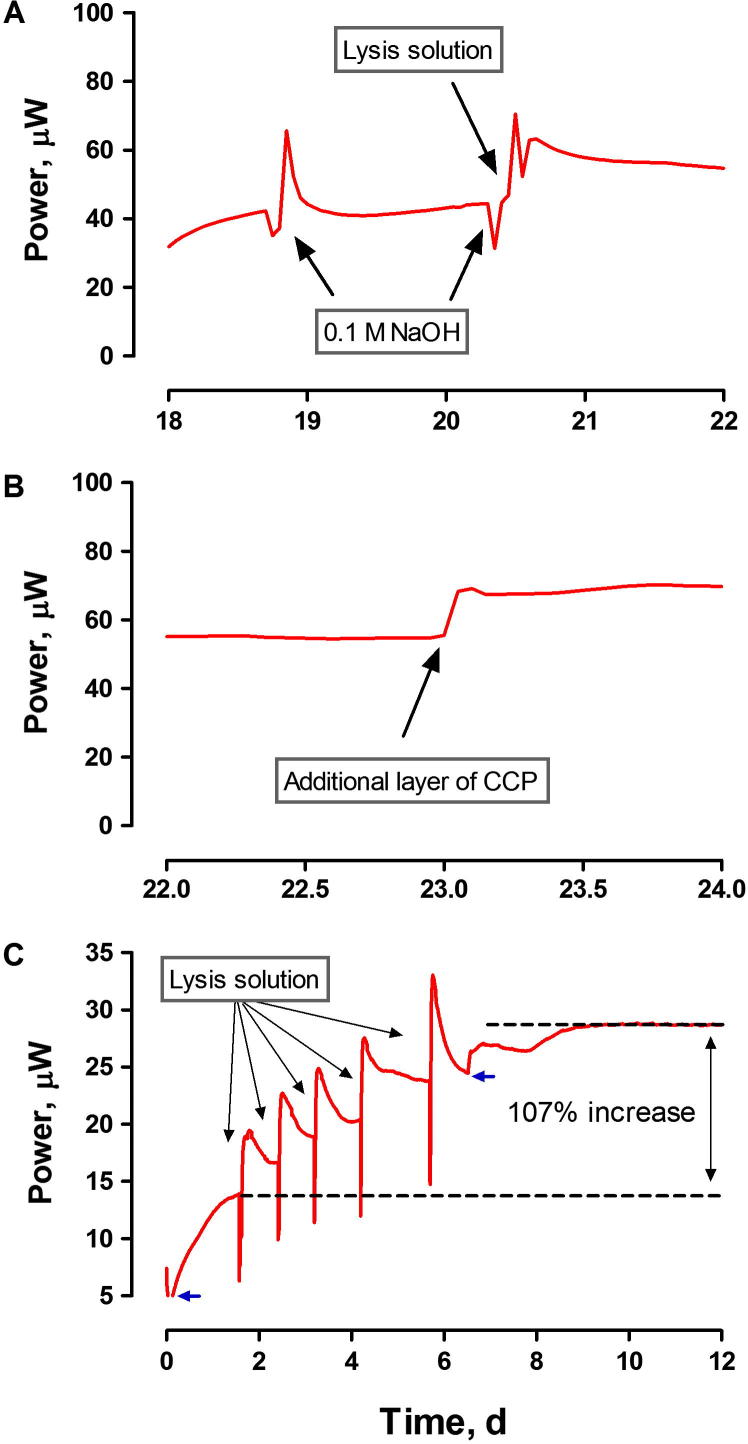
Temporal effect of regeneration steps on power performance of an individual MFC. A – Step 1: addition of the alkaline lysis solution. B – Step 2: replacing the outer layer with the same loading of carbon. C – Power output improvement from sequential treatment of the cathodic biofilm with chemical lysis; data are from a separate experiment under batch conditions. Blue arrows indicate points of feeding with urine.

To bring the MFC performance back to the original level, two regeneration steps were performed. Applying the 0.1 M NaOH solution resulted only in a temporary increase in perfomance. Increasing the lysis kinetics by increasing the NaOH concentration, raising the solution temperature to 60 °C as well as adding the surfactant, resulted in a more stable increase of power performance (Fig. 4). The NaOH and Triton X-100 solution helped to break down the bacterial cell membranes [35], resulting in the suppression of the cathodic biofilm activity. The presence of the surfactant solution has also probably helped to remove the chemical compounds adsorbed to the carbon electrode surface. The removal of the biofilm and scale from the cathode surface was confirmed by the ESEM analysis (Fig. 3). An alternative procedure, consisting of sequential treatment of the cathode with the lysis solution, resulted in an increase in power performance by 107% (Fig. 4). In batch conditions (in which the anolyte is not constantly being replenished by fresh feedstock) the lysis solution could be toxic to the biofilm, if its diffusion to the anodic chamber occurred. Poisoning the anode would cause a decrease of the overall MFC performance. Nevertheless, this effect was not observed either in batch or continuous flow conditions, indicating that this chemical regeneration procedure can be safely used, during the operation of bioelectrochemical systems. These results also suggest that application of the lytic solution before such a significant power decrease was observed, could prevent deterioration of the cathodic material and thus the need for replacing the outer layer of the cathode (the second step of proposed procedure).

The lysis solution consisted of sodium hydroxide, which is a commercially available product. For real world applications, an alkaline solution could easily be delivered as a commercially available chemical, or even by developing methods for its bioelectrosynthesis in MFCs. Such a sustainable way of alkali production was demonstrated by Gajda et al. [36].

**Fig. 5:**
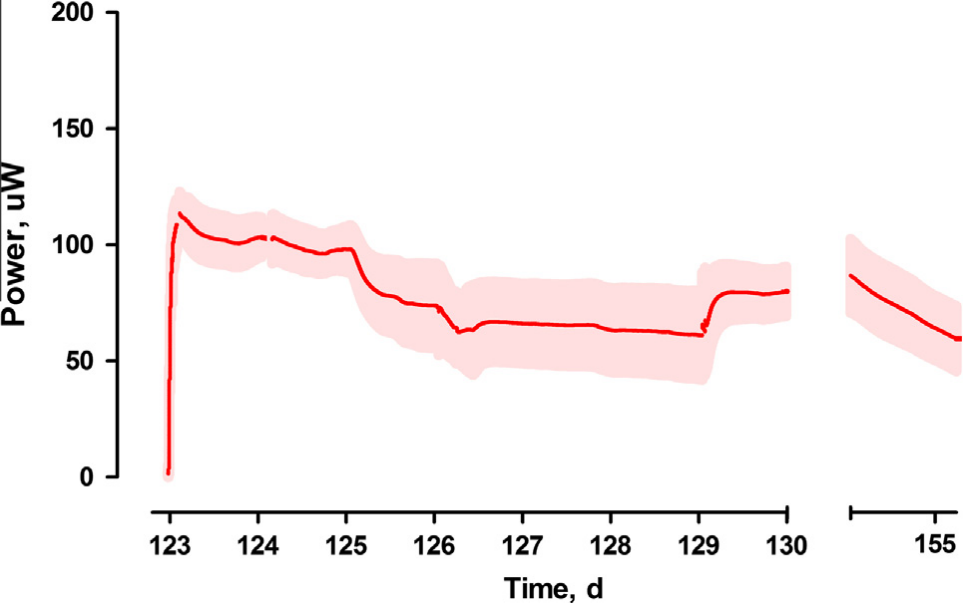
Temporal performance of MFCs after the removal of the cathodic biofilm. Data represent average ± standard deviation. Data recorded for all MFCs in the cascade.

Removing the external layer of cathode and re-painting it with a new layer of CCP resulted in a further increase in power output, suggesting that the biofilm has also caused physical blockage or deterioration of the cathodes. This two-step cathode regeneration resulted in 100% recovery of the power performance to the original levels: 105.3 ± 16.3 μW (Fig. 5).

**Fig. 6:**
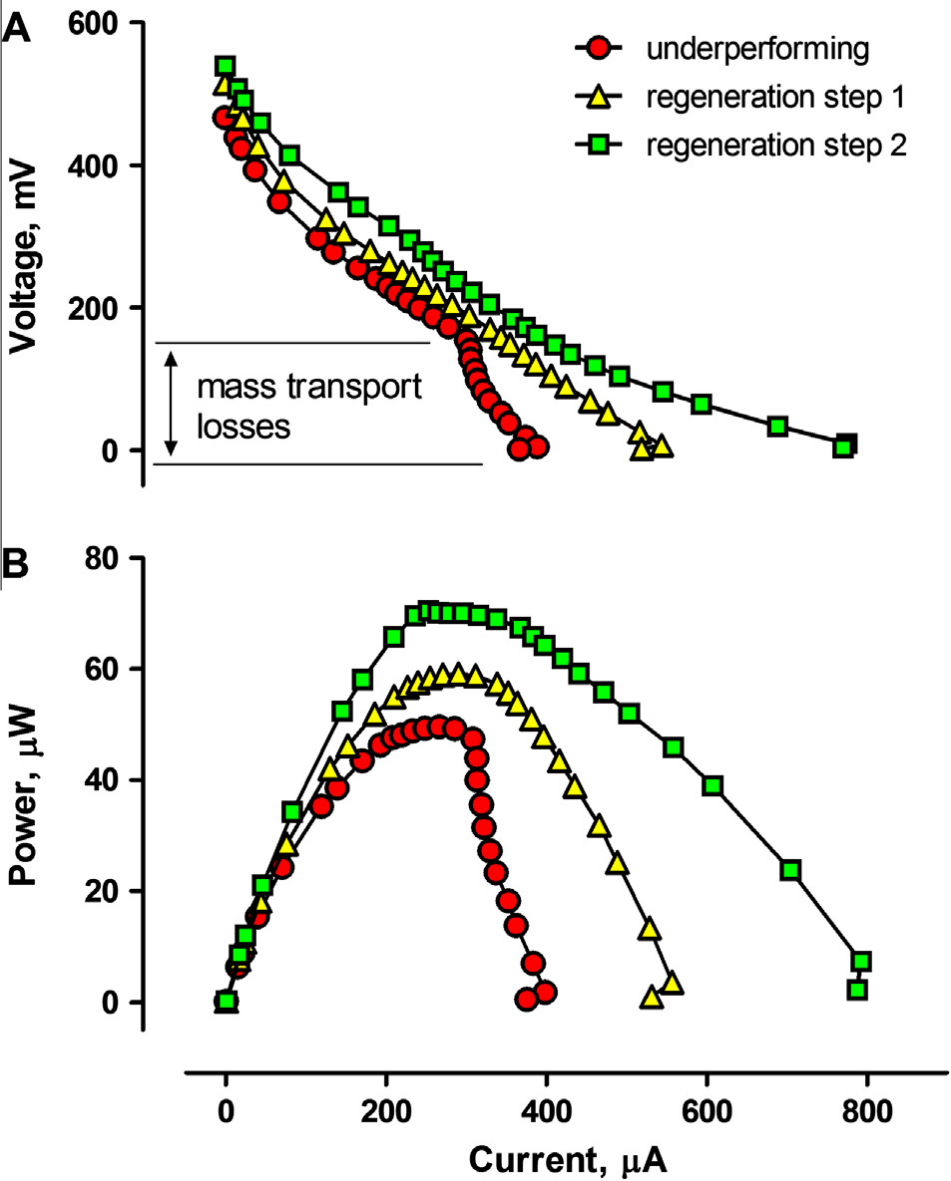
Polarisation (A) and power (B) curves for MFCs before and after the regeneration steps. The maximum power observed during polarisation (70.1 μW) was lower than that observed in real time (105.3 ± 16.3 μW – corresponding to 100% recovery) due to the variance in fresh urine composition.

The findings derived from real time performance monitoring were also confirmed by polarisation experiments (Fig. 6). The polarisation experiments of the MFCs operating for 86 days, indicated that the deterioration in performance was mainly caused by the mass transport losses. For the example given, treating the cathodic surface with the lysis solution resulted in an increase in current from 398.1 to 556.9 μA and power from 49.6 to 59.1 μW. The replacement of the external CCP layer resulted in a further increase of current and power to 792.4 μA and 70.2 μW, respectively. Similarly, the open circuit potential of the MFCs was also affected by the biofilm formation, whereby following the recovery procedure the open circuit voltage increased from 466.5 to 514.8 and 539.2 mV, respectively. The power output recorded during the polarisation experiments was lower than the real time performance recorded subsequently. It is assumed that the variation was caused by the composition of human urine which varies, depending on the type of diet of consenting donors. The values indicating the efficiency of power recovery are summarised in Table 1.

### 3.3 Removal of the biofilm during regeneration

**Fig. 7:**
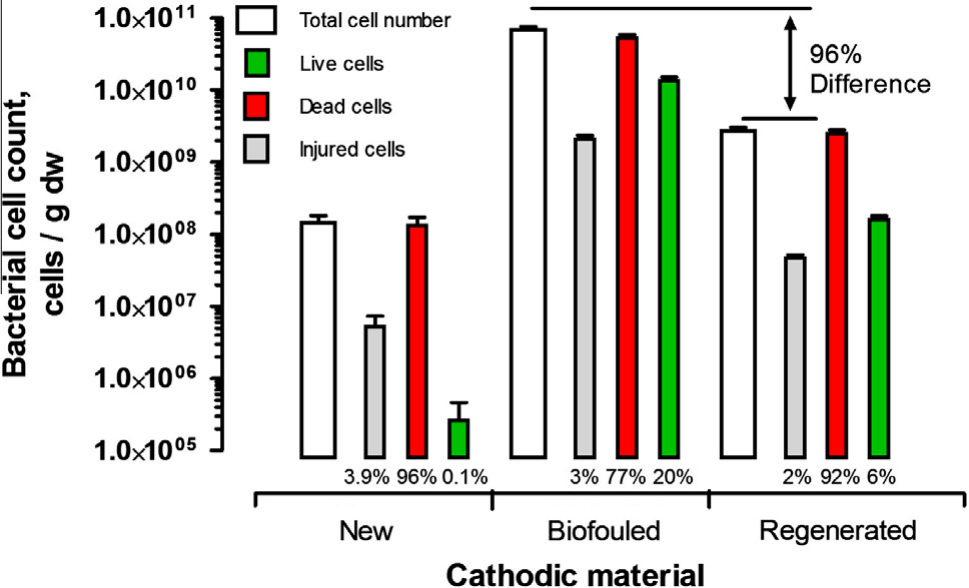
Efficiency of the regeneration procedure in removing the living and dead bacterial biomass from the biodeteriorated cathode. New – MFC operating for 2 weeks; biofouled – MFC operating for 3 months; regenerated – MFC operating for 1 month following regeneration. The percentage values are indicating the contribution of subpopulations in the total bacterial population. Samples were taken from the 2^nd^ MFC in the cascade.

Monitoring of the biofilm growth at the cathode surface over the experimental period was conducted with flow cytometry and viable cells staining (Fig. 7). The results indicated that biofilm had started to develop at the cathode surface in the early stages of operation of the MFCs (14 days), for cathodes which were considered to be new. Total bacterial count observed for this period was 1.5 ∗ 10^8^ cells / g dw. However, the total biomass was composed of only 0.1% of living, active bacterial cells. Prolonged operation of MFCs led to a significant increase of both total and viable bacterial biomass. The total bacterial cell count increased by two orders of magnitude and was composed of 20% of living and respiratory bacterial cells. The respiration of the living bacteria was competitive to spontaneous cathodic reaction occurring at the cathode surface, thus causing the cathode overpotential.

The regeneration procedure and subsequent 1-month operation of the regenerated cathode, led to a 96% decrease of the total bacterial biomass and significantly decreased the viability of bacteria that formed the biofilm (to 6%). The contribution of injured bacterial cells in the overall population was stable and reached 2–3.9%. The occurrence of injured cells may have been caused by several factors, including environmental, storage and staining conditions.

Other studies showed that mass transport losses in MFCs may be caused by the decrease of oxygen diffusion rates to the cathode surface, which could be caused by a biofilm growth on the cathodic inner layer. This has been reported as an advantage since it can potentially decrease the oxygen crossover to the anodic chamber [28]. Excessive biofilm growth on the inner cathode layer may be controlled by applying polymeric electrode separators [37]. However, during the operation of MFC-based systems, events such as leakages, overflow or electroosmotic drag may also result in biofilm formation on the external layer of the cathode and cause the reduction of the power performance as has been demonstrated in this study. The possible solution for such occurrences could be the development of physical and chemical *in-situ* regeneration methods for carbon based cathodes and the incorporation of multiple cathode layers. These failsafe mechanisms need to retain the anodic half-cell integrity, as proposed in this work.

## 4 Conclusions

The power performance of the air–cathode MFCs decreased after three months of operation by one order of magnitude in comparison to their initial performance. The decrease of absolute power was caused by the biofilm formed on the outer layer of the carbon painted cathodes. The easy and inexpensive way to regenerate the MFC performance *in-situ* was adapted from the molecular biology method for lysis and extraction of biomass, and its use resulted in improving the performance. The additional step of replacing the external layer of the cathode resulted in returning the performance to the original level, reaching 100% recovery. The monitoring of the biofilm growth indicated that biofilm started to form at the early stages of operation. The regeneration procedure proposed in this study leads to a significant decrease of bacterial biomass and of its viability. These results indicate that regenerating the biofouled cathodes by applying the lysis solution could be an appropriate tool for preventing or removing undesirable biofilm growth. The advantage of the proposed procedure, for *in-situ* chemical regeneration of the cathode, is its simplicity. The proposed protocol of chemical regeneration of the carbon-based cathodes, may form an integral part of future scaled-up MFC systems running in real world environments, for on-site & on-line system maintenance.

## Figures and Tables

**Table 1:** Summary of the results from the regeneration procedure. The recovery of performance as a percentage is given in parentheses. Values in bold represent the average of all MFCs in the cascade.

Experiment type	Data origin	Figure #		Power (μW)			
Performance at start up	Fouling (before treatment)	After 1st step of regeneration (alkaline lysis)	After 2nd step of regeneration (additional CCP layer)	Recovery of initial performance
Batch mode MFC	Real time	Fig. 4	–	13.5	28.6 (107%)	–	–
Continuous flow/individual MFC	Real time	Fig. 4	–	44.2	63.3 (43%)	68.3 (54%)	–
Continuous flow/individual MFC	Polarisation	Fig. 6	–	49.6^a^	59.1 (19%)^a^	70.5 (40%)^a^	–
Continuous flow/cascade of MFCs	Real time	Fig. 2, Fig. 5	**104.9** **±** **32.2**	**13.3**	–	**105.3** **±** **16.3 (791%)**	**100%**

a Before regeneration MFCs were fed with a different batch of fresh human urine than during regeneration, which caused lower power recovery during polarisation, in comparison with the real time data, which corresponded to 100% recovery.
